# Prostate-Centric Versus Bony-Centric Registration in the Definitive Treatment of Node-Positive Prostate Cancer with Simultaneous Integrated Boost: A Dosimetric Comparison

**DOI:** 10.1016/j.adro.2022.100944

**Published:** 2022-03-16

**Authors:** Trudy C. Wu, Michael Xiang, Nicholas G. Nickols, Stephen Tenn, Nzhde Agazaryan, John V. Hegde, Michael L. Steinberg, Minsong Cao, Amar U. Kishan

**Affiliations:** a Department of Radiation Oncology, University of California, Los Angeles, Los Angeles, California; b Department of Radiation Oncology, VA Greater Los Angeles Healthcare System, Los Angeles, California; c Department of Urology, University of California, Los Angeles, Los Angeles, California

## Abstract

**Purpose:**

To determine the effect of daily shifts based on rigid registration to intraprostatic markers on coverage of boost doses delivered to gross nodal disease for prostate cancer.

**Methods and Materials:**

Seventy-five cone beam computed tomographies (CBCTs) from 15 patients treated with definitive radiation for clinically node-positive prostate cancer underwent fiducial-based and pelvic bony-based registration to the initial planning scans. Gross tumor volumes of nodal boost targets were contoured directly on each CBCT registration. The nodal displacement (3-dimensional translation from the node centroid on planning CT to node centroid on registered CBCT) and dose coverage (minimum dose [Dmin], mean dose [Dmean], dose delivered to 95% of the gross tumor volumes [D95]) were calculated for each registration on all nodal targets. All doses for each node were normalized to its intended prescription dose (dose covering 95% of a 3 mm planning target volume [PTV] expansion).

**Results:**

Forty-one gross nodal targets were analyzed. Most boosted nodes (80.5%, 33/41) were treated with conventional fractionation using volumetric-arc radiation therapy, and 19.5% (8/41) underwent stereotactic body radiation therapy (SBRT). Dmin, Dmean, and D95 were all significantly lower with fiducial-based registration compared with bony-based registration (*P* < .0001). Nodal displacement was significantly higher for fiducial-based registrations (*P* < .0001). The 3-dimensional translation between the fiducial-based and bony-based registrations (bony-to-fiducial vector) was the most significant predictor of nodal displacement (*P* < .0001). On fiducial-based registrations, a 3 to 5 mm gross nodal PTV margin is sufficient in most directions; however, superior and posterior margins of 8 to 9 mm are required as a result of asymmetrical prostatic motion.

**Conclusions:**

Large and anisotropic PTV margins are likely needed to adequately dose gross nodal targets when patient setup is based on rigid registration to intraprostatic markers. Alternative approaches such as adaptive replanning may be required to overcome these limitations.

## Introduction

The preferred treatment for patients with clinically node-positive prostate cancer is external beam radiation therapy with concomitant androgen deprivation therapy.^1^ Radiation planning volumes include the prostate, seminal vesicles, and pelvic lymph node basin with a simultaneous integrated boost (SIB) to gross nodal disease detected on pretreatment imaging. Image guided radiation therapy (IGRT) for daily patient setup is typically accomplished using rigid registration (RR) to intraprostatic markers (IPMs) via orthogonal kilovoltage x-ray imaging or cone beam computed tomography (CBCT). However, due to independent movement between the prostate and lymph nodes, a persistent concern has been that aligning to the prostate might lead to underdosing of nodal targets.^2^

The dosimetric effect on elective pelvic node coverage with conventional fractionation has been investigated and found to be negligible, largely because the effect of motion may vary randomly along any individual axis, such that interfractional shifts may counterbalance themselves.^3^^,^^4^ In a condensed treatment regimen like stereotactic body radiation therapy (SBRT), such a counterbalance may not occur, as there are far fewer treatments. However, even in patients treated with SBRT, elective pelvic nodal clinical target volume dosing is generally maintained, with >97% of the prescribed dose delivered if superior-inferior axis translations and bladder height changes are kept minimal.^5^^,^^6^ Recently, the advent of advanced molecular imaging studies (eg, prostate-specific membrane antigen [PSMA] positron emission tomography [PET]) has enabled detection of occult nodal disease at presentation which may be amenable to a gross nodal boost.^7^, ^8^, ^9^

As numerous studies have demonstrated, the prostate is a highly mobile organ and can result in interfraction variations of 1.52 and 1.45 cm in the SI and AP axes, respectively.^10^, ^11^, ^12^, ^13^^,^ On the other hand, previous analysis showed that aligning to IPMs still maintained adequate coverage of elective nodal volumes in most patients*.*^5^ Because prostatic motion is known to be large in some cases and independent of pelvic lymph nodes, it is still unclear whether daily shifts based on RR to IPMs may compromise the boost dose delivered to gross nodal disease, and if so, to what extent.^14^ Furthermore, planning target volume (PTV) expansions for nodal boosts generally require tighter margins than elective nodal regions, due to the proximity of critical organs at risk (OARs) and a higher prescription dose. Such smaller margins may further jeopardize dosimetry to gross nodal targets, and underdosing gross disease, whether prostatic or nodal, may compromise the probability of curative outcome. Thus, evaluation of dose delivery to gross nodal targets is critical to understand whether our current treatment planning techniques are satisfactory and meet expectations.

The objectives of this study are to (1) determine how relative motion between the prostate and lymph nodes effect dose delivery to gross nodes; and (2) to evaluate how daily IGRT, treatment planning, and dosimetry can be improved to achieve optimum dose coverage.

## Methods and Materials

### Patient cohort

The study cohort comprised of 15 patients treated with definitive radiation for clinically node-positive prostate cancer at a single institution from 2018 to 2020. All patients had evidence of nodal involvement on staging CT or PSMA PET and were treated with a boost to gross lymph nodes. Gross nodal targets were contoured and expanded 3 mm in all directions to form the PTV volume. Lymph nodes located beyond the superior CBCT scanning limit (ie, upper common iliac or para-aortic nodes) were omitted from this analysis (29.3% [17/58] of lymph nodes from 60.0% [9/15] of patients). Fourteen patients were treated with TrueBeam (Varian Medical Systems, Inc, Palo Alto, CA) and one with NovalisTx (BrainLab AG, Munich, Germany). Each CBCT was matched to fiducials before treatment delivery. Institutional review board approval was in place for this study.

Eleven patients (73.3%, 11/15) underwent a fractionated course of volumetric modulated arc therapy (VMAT), of which 10 received a nodal boost via SIB to 62.5 Gy in 25 fractions. In one patient, gross nodal targets were boosted sequentially to 61.2 Gy in 34 fractions. Four patients were treated with SBRT (5 fractions) and received boost doses via SIB from 35 to 40 Gy. A total of 41 gross nodal targets were included in the data analysis, with a median of 2 nodes per patient (range, 1-8 nodes per patient). The majority of boosted nodes were treated in patients receiving fractionated VMAT (80.5%, 33/41) and prescribed a median nodal dose (PTV D95) of 61.6 (interquartile range [IQR] 60.3-62.1) Gy. Primarily, nodes were located in the external iliac (41.5%, 17/41) and internal iliac (26.8%, 11/41) chains with a median node-to-prostate distance of 8.5 (IQR 7.3-9.8) cm on planning CT. Additional nodal characteristics are outlined in Table 1.

**Table 1:** Nodal characteristics

	N = 41
Location	
Common iliac	5 (12.2%)
External iliac	17 (41.5%)
Internal iliac	11 (26.8%)
Obturator	5 (12.2%)
Perirectal	1 (2.4%)
Presacral	2 (4.9%)
Fractionation	
SBRT	8 (19.5%)
Conventional VMAT	33 (80.5%)
Prescription dose (PTV D95)*	
SBRT	36.0 Gy (35.7-36.5)
Conventional VMAT	61.6 Gy (60.3-62.1)
Node-to-prostate distance*	8.5 cm (7.3-9.8)
GTV*	0.5 cc (0.3-1.2)

⁎ Median (interquartile range). *Abbreviations*: D95 = dose delivered to 95% of the gross target volume; GTV = gross target volume; PTV = planning target volume; SBRT = stereotactic body radiation therapy; VMAT = volumetric-arc radiation therapy.

### Procedures

Five CBCT scans were analyzed for each patient, resulting in a total of 75 CBCTs for analysis. For patients undergoing treatment with conventional fractionation, 1 CBCT per 5 fractions was selected (ie, CBCT from fraction 1, fraction 6, etc). For patients receiving SBRT, the CBCT from each fraction was used. Each CBCT was uploaded into MIMVista (MIM Software, Inc, Cleveland, OH) and aligned to the planning CT simulation scan using 2 methods. First, an entirely prostate-centric fiducial-based registration was performed using 3 degrees of freedom (DOF) via rigid registration to IPMs. Second, an entirely pelvic node-centric registration was performed using 6 DOF via rigid registration to pelvic bony-based anatomy. Our choice of 3 DOF for prostatic fiducials and 6 DOF for pelvic CBCT reflects a pragmatic study design that emulates and compares the 2 most common clinical workflows. The gross tumor volumes (GTV) of nodal boost targets were then contoured directly on each CBCT registration by 2 investigators (TCW and MX). The nodal displacement was calculated as the 3-dimensional translation from the node centroid on planning CT to node centroid on the registered CBCT, and analyzed in 3 spatial axes (left to right, anterior to posterior [AP], superior to inferior [SI]). The original dose distribution was overlayed on each fiducial-based and bony-based CBCT registration. The dose coverage, as measured by the minimum dose (Dmin), mean dose (Dmean), and dose delivered to 95% of the GTV (D95), was then calculated for both registrations on all individual lymph nodes. All doses were normalized to the intended prescription dose on a per-node basis, which was defined as the D95 of the nodal PTV on the planning scan.

The bony-to-fiducial vector was the 3-dimensional translation between the fiducial-registered CBCT and the pelvic bony-registered CBCT.^5^ Additional study variables were the rotations imposed by the 6 DOF pelvic registration, distance from each node centroid to the prostate centroid on the planning CT scan, size of the nodal GTV, and nodal location (eg, internal iliac, external iliac, obturator, presacral). The bladder height (maximum longitudinal measurement on the anterior-most coronal plane containing the prostate) and rectal diameter (maximum transverse measurement at midprostate) were recorded for each planning CT scan and CBCT. All patients at our institution are instructed to have a full bladder and empty rectum before treatment, which is particularly emphasized for patients undergoing SBRT.

### Statistical analysis

The Wilcoxon signed-rank test was used to compare paired differences for coverage and centroid displacement between fiducial-registered CBCTs and pelvic bony-registered CBCTs. Spearman's rank correlation was used to evaluate the associations between coverage, nodal displacement, and magnitude of the bony-to-fiducial vector. Additionally, due to the hierarchical structure of the data, ie, 5 CBCTs per node and (in some cases) multiple nodes per patient, linear mixed-effects models were used to analyze the relationships between coverage, nodal displacement, and other study variables, allowing for random effects per-node and per-patient. PTV margin calculations were performed using the classic van Herk formula, based on the systematic and random errors, as previously described.^10^^,^^15^ MATLAB version R2020a (MathWorks, Inc., Natick, MA) was used for calculations. All tests were 2-sided and considered significant at *P* < .05.

## Results

**Fig. 1:**
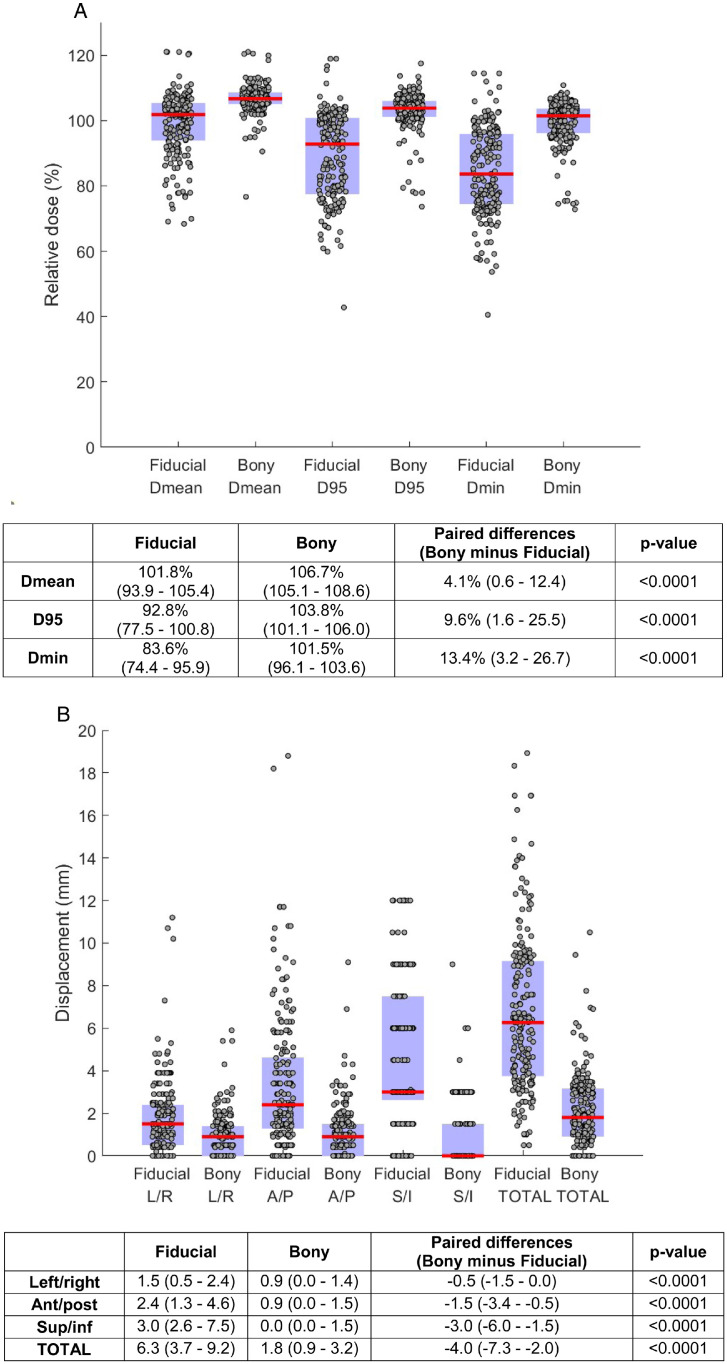
Comparison of fiducial-registered versus pelvic bony-registered cone beam computed tomographs for A, dose coverage and B, nodal displacements relative to the planning computed tomography. Each data point represents 1 node on 1 cone beam computed tomography. Red bars in the figure and primary values in the table represent medians. Blue patches in the figure and parenthesized values in the table represent interquartile ranges. *Abbreviations*: Ant/Post = anterior to posterior; AP = anterior to posterior; Dmean = mean dose; Dmin = minimum dose; D95 = dose delivered to 95% of the gross target volume; LR = left to right; SI = superior to inferior; Sup/inf = superior to inferior.

Dose coverage to gross nodal targets was significantly lower for fiducial-based registration compared with pelvic bony-based registration for all dose metrics (Dmean, D95, Dmin; all *P* < .0001) relative to the intended prescription dose (Fig 1A). Similar results were obtained when CBCTs were analyzed in aggregate per node, and when nodes were analyzed in aggregate per patient (Figs E1-2).

**Fig. 2:**
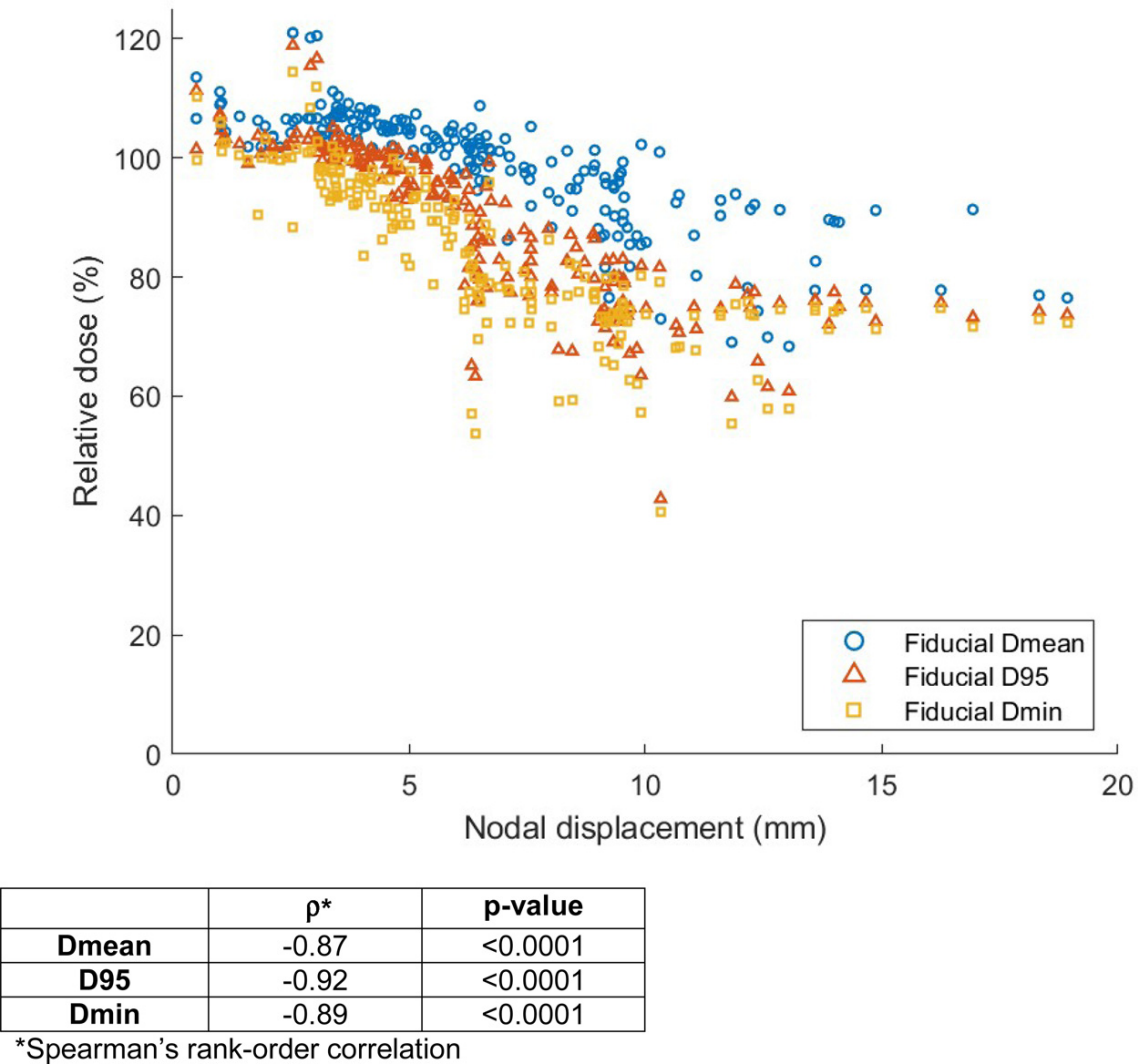
Relationship of nodal displacement and dose coverage. *Abbreviations*: D95 = dose delivered to 95% of the gross target volume; Dmean = mean dose; Dmin = minimum dose.

To investigate the cause of the disparity in dose coverage, we analyzed the displacement of gross nodal targets on the registered CBCTs compared with the planning CT. Nodal displacement was significantly higher for fiducial-based registrations compared with pelvic bony-based registrations in all 3 spatial axes (left to right, AP, SI) and overall (all *P* < .0001; Fig. 1B). In total, the nodal displacement was a median of 4.0 (IQR 2.0-7.3) mm greater for fiducial-based registrations compared with pelvic bony-based registrations. Similar results were obtained when CBCTs were analyzed in aggregate per node, and when nodes were analyzed in aggregate per patient (Figs E3-4). Dose coverage was highly significantly negatively correlated with nodal displacement (*P* < .0001 for all dose metrics), suggesting that increased nodal displacement was the driver of reduced coverage in the fiducial-based registrations (Fig 2, Table E1).

Next, we investigated factors that predicted for increased nodal displacement on the fiducial-based registrations using linear mixed-effects models (Table 2). The magnitude of the bony-to-fiducial vector was highly positively correlated with the total nodal displacement and was the major explanatory variable, with a 0.6 mm increase in nodal displacement for every 1.0 mm increase in bony-to-fiducial translation (*P* < .0001; Table 2, Fig E5). Daily changes in bladder height and rectal diameter were not found to be predictive of nodal displacement.

**Table 2:** Predictors of nodal displacement

Variable	Effect (95% CI)	*P* value
Bony-to-fiducial vector	0.6 mm per 1 mm (0.5-0.8)	< .0001
Node-to-prostate distance	0.2 mm per 1 cm (–0.0 to 0.1)	.36
Location		
Common iliac	Reference category	—
External iliac	–0.1 mm (–2.8 to 2.6)	.93
Internal iliac	–0.9 mm (–3.8 to 2.0)	.55
Obturator	–2.5 mm (–5.0 to –0.0)	.05
Perirectal	–1.5 mm (–4.5 to 1.6)	.33
Presacral	1.3 mm (–4.7 to 7.3)	.67
GTV	–0.6 mm per 1 cc (–1.1 to –0.1)	.01
Axial rotation	0.0 mm per 1 degree (–0.9 to 0.9)	.96
Sagittal rotation	0.5 mm per 1 degree (0.0-0.9)	.05
Coronal rotation	–0.3 mm per 1 degree (–0.9 to 0.3)	.33
Rectal diameter	–0.1 mm per 10% (–0.6 to 0.3)	.56
Bladder height	0.1 mm per 10% (–0.0 to 0.2)	.26

*Abbreviations*: CI = confidence interval; GTV = gross target volume.

**Fig. 3:**
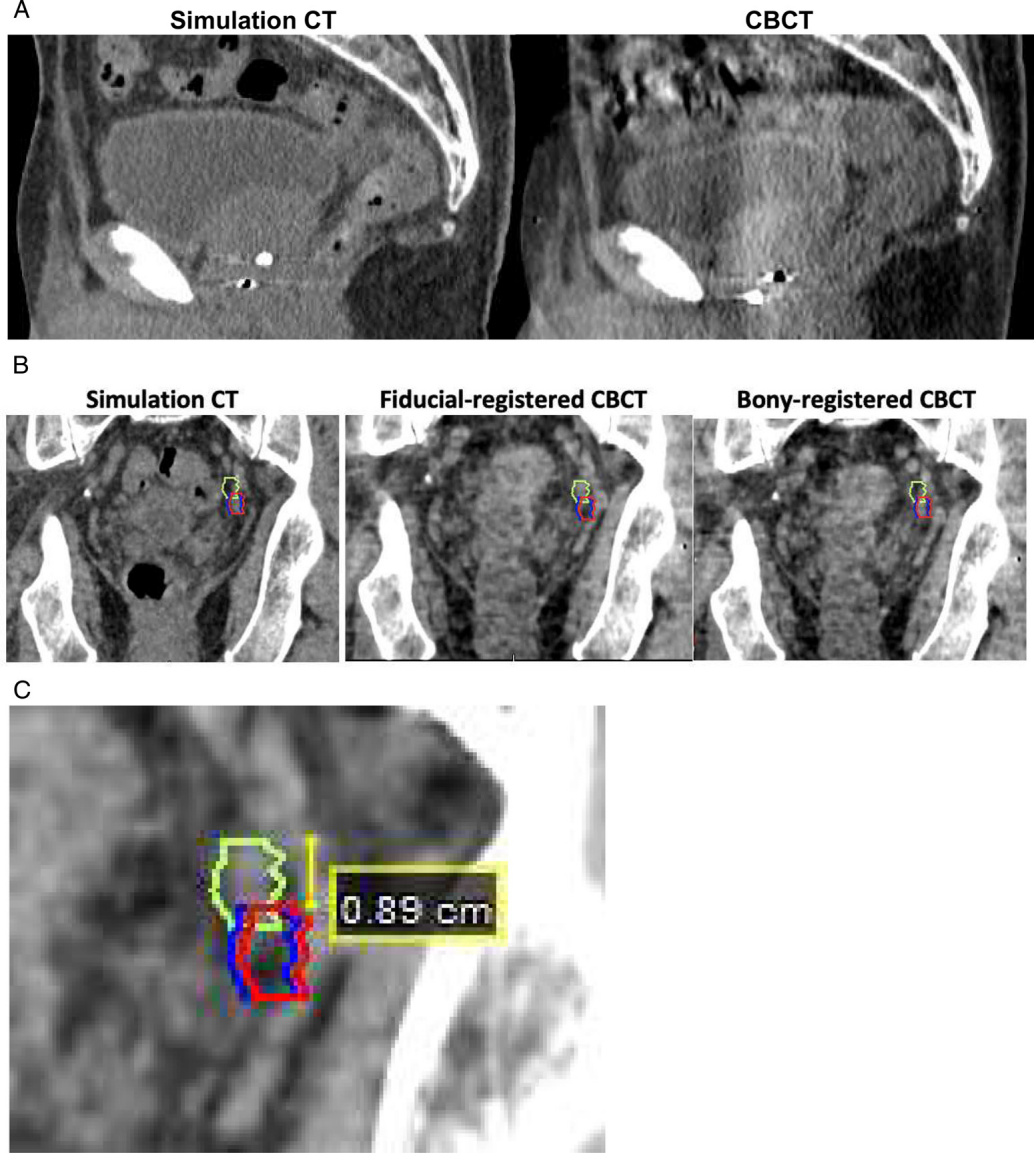
Example of a patient with a large inferior prostatic motion at treatment compared with planning computed tomography (CT) and the effect on positioning of the gross nodal target. A, Sagittal view of the simulation CT (left) and cone beam CT (CBCT; right); note the relative positioning of the intraprostatic fiducial markers relative to the pubic symphysis. B, Gross nodal target (left internal iliac lymph node) contoured on the simulation CT (red), fiducial-registered CBCT (lime green), and pelvic bony-registered CBCT (blue), overlaid on the simulation CT (left), fiducial-registered CBCT (middle), and bony-registered CBCT (right). C, A 9-mm superior planning target volume margin on the gross nodal target would be required to encompass the actual position of the node on the fiducial-registered CBCT.

Finally, we analyzed the extent of PTV margins that would be required to ensure intended dose coverage on fiducial-based registrations. Using a cumulative histogram (Fig E6), a 3 to 5 mm isotropic margin would encompass >90% of nodal displacements in all directions found in this study, except for superior and posterior, for which nodal displacements were larger. In the posterior direction, a 6 mm margin was required to cover >80% of nodal displacements, and an 8 mm margin was required to cover >90%. In the superior direction, an 8 mm margin covered 76% of nodal displacements, and a 9 mm margin covered >90%. Similar results were obtained using the van Herk formula (Table E2). An illustrative case example is provided in Fig 3, in which there was a large inferior motion of the prostate at treatment compared with the planning CT. Consequently, a greater superior PTV margin on the gross nodal target would have been required to ensure that it was covered on the fiducial-registered CBCT.

## Discussion

We compared the outcomes of fiducial-based registration (an entirely prostate-centric approach to alignment) versus pelvic bony-based registration (an entirely nodal-centric approach to alignment) on gross nodal coverage and displacement. Our study reveals that dose coverage was significantly lower, and nodal displacement was significantly greater, for fiducial-based registration compared with pelvic bony-based registration. Our results have implications on the feasibility of dose escalation to clinically positive nodes for prostate cancer and the choice of treatment approach or technique.

Most commonly in the modern era, daily IGRT uses CBCT or orthogonal x-ray alignment to IPMs in patients undergoing definitive treatment for prostate cancer.^16^, ^17^, ^18^ Previously, dose delivery to elective nodal volumes (in clinically node-negative patients) using RR to IPMs has been well-studied, and was found to achieve the adequate clinical coverage in both conventional and ultrahypofractionated regimens in most situations.^3^^,^^5^^,^^6^ In contrast, our study reveals that gross nodal targets in clinically node-positive patients are far more sensitive to small shifts, resulting in significant undercoverage when aligning to IPMs. This failure at dose delivery can be attributed to a larger relative shift in the setting of small lymph node boost volumes and a steeper dose drop off surrounding nodal boost targets. Although alignment to IPMs leads to significantly lower coverage of gross nodes compared with bony-based registration, the Dmean was still found to be reasonably acceptable at >90% in most cases. However, the D95 and Dmin could drop as low as 75% to 80%. Of note, the surrounding elective nodal volume was dosed to 45 Gy in patients receiving fractionated treatment with integrated boost to approximately 60 Gy; thus, the Dmin could not be lower than approximately 75%, even if the lymph node was completely off-target, as long as it remained within the elective nodal volume. Dmean, D95, and Dmin were inversely correlated with nodal displacement, but the relationship was not strictly linear, as the rate of dose fall-off around the PTV is also not linear, and may also depend on additional factors such as conformality, and location of nodal boost targets relative to the elective nodal volume and the high-dose prostate volume.

Nodal coverage was tightly correlated with nodal displacement, and the largest predictor of nodal displacement was the magnitude of the bony-to-fiducial vector. This vector is mainly a surrogate and direct measure for prostatic motion, as it reflects the 3-dimensional translational shift between alignment to the prostate via IPMs relative to pelvic bony anatomy. Our results redemonstrate this high degree of prostatic mobility, which unfortunately, cannot be reliably predicted or controlled. To ensure intended dose coverage to nodal targets while aligning to IPMs, large PTV margins would be required, specifically in the superior and posterior directions. We speculate that one possibility is that, on average, the “resting” (or physiological) location of the prostate tends to be more superior and posterior anatomically, but it occasionally makes large excursions in the inferior or anterior directions, which was detected on CBCT and was responsible for driving the larger observed nodal displacements in the superior and posterior directions. By contrast, no such preference was observed in the left versus right directions, which is consistent with humans being bilaterally symmetrical. The expansion of margins we are suggesting for gross nodal boost targets to 8 to 9 mm are consistent with consensus guidelines for elective nodal volumes.^19^ Thus, our results reflect the inherent reality of prostatic motion as an impediment to delivering high doses both to the prostate (which can move substantially) and gross nodal targets (which are relatively immobile with respect to bony anatomy). Patients treated with prostate radiation therapy are instructed to undergo radiation with a full bladder and empty rectum to limit dose to OARs. To some extent, these maneuvers might also be expected to ensure more consistent daily setups and decrease prostatic motion. However, in our study, nodal displacement was not significantly correlated with changes in bladder or rectal filling, again suggesting that the majority of nodal displacement is due to stochastic prostatic motion.

We herein propose several potential approaches to improve dose coverage of gross nodal targets, although each has associated limitations. First, one solution is to increase gross nodal PTV margins in the superior and posterior directions while continuing to align purely to IPMs, to compensate for asymmetrical prostate motion favoring the inferior and anterior directions, respectively. However, the extent of PTV expansions in these directions may be prohibitively high when considering nearby OARs (ie, bowel). Second, rather than aligning purely to the IPMs, one could align the patient to a combination of pelvic bony anatomy and IPMs. However, this may compromise dose delivered to the prostate and can be challenging to ensure both the prostate and nodal targets are within their respective PTVs unless one or both PTVs are expanded.^4^^,^^20^, ^21^, ^22^ Third, different prostatic shifts can be modelled and planned for in advance, with an appropriate plan being selected at the time of treatment based on the “prostate position of the day.”^2^ However, the main drawback of this approach is the time and labor-intensive effort to generate a large library of contingency radiation plans. Fourth, additional dose to gross nodal targets (with or without elective lymph node volumes) can be delivered as a sequential boost using a second, separate plan with the patient aligned to pelvic bony anatomy. Drawbacks of this approach include longer treatment times and dosimetric uncertainties due to variations in overlap. Fifth, advanced radiation technologies using adaptive replanning can be used to develop a customized treatment per fraction based on the “anatomy of the day.” However, this requires specialized equipment, prolongs the treatment time, and the prostatic position may change again during the course of replanning.^2^

A strength of this study is our focus on individual gross nodal targets receiving a boost, whereas prior studies did not examine individual gross nodal targets or quantify the effect to their dosimetric coverage.^22^^,^^23^ In a study by Kershaw et al, a role for larger PTV margins was also described, specifically in the SI and AP axes, with prostate-centric registration to ensure better setup of the entire lymph node basin (not individual nodal targets). The pelvic lymph node clinical target volume was further subdivided into right to left and external to internal iliac regions and there was no significant difference in the 4 lymph node regions with prostate-centric or bony-centric registration. For a 3 DOF couch, Kershaw et al recommended a 9 mm and 5 mm margin in the AP and SI directions, respectively. Use of a 6 DOF couch allowed for further margin reduction in the AP axis alone (from 9-6 mm). However, a dosimetric analysis was not performed in the prior study.^20^

We recognize several limitations apply to this study. First, 17 lymph nodes from 9 patients were outside of the CBCT field of view and were thus excluded from our analysis. Therefore, we were unable to evaluate dose delivery to lymph nodes further away from the prostate. However, we did not find a significant relationship between nodal displacement and node location or distance between the node and the prostate. Second, as only 8 lymph nodes (from 4 patients) were treated with SIB using SBRT, it is difficult to draw meaningful conclusions on whether dose delivery to such small targets is further compromised with ultrahypofractionation. It is conceivable that the dosimetric effect for conventionally fractionated patients is less because any misalignments are spread out over more fractions^3^^,^^4^; however, our analysis showed that undercoverage was highly significant even on a per-patient basis, suggesting that treatment over a larger number of fractions (or examining more CBCTs) would not change our findings. Lastly, it is unclear what effect the dosimetric undercoverage, to the degree found in this study, has on clinical outcomes such as biochemical recurrence; the Dmin was affected most and Dmean was affected least. There is also uncertainty regarding the dose required to sterilize gross nodal targets, which may also depend on lymph node size. At a minimum, however, our study should alert clinicians to the possibility that gross nodal targets are being significantly underdosed relative to the physician's intent.

## Conclusions

Dose escalation to gross nodal targets using a narrow isotropic PTV margin has a high risk of undercoverage when patient setup is based on rigid registration to intraprostatic markers. Alternative approaches, such as increased PTV margins or adaptive replanning, may be required to overcome these limitations.

## Appendix Supplementary materials

Image, application 1
