# Supplementary material for: Prostate-Centric Versus Bony-Centric Registration in the Definitive Treatment of Node-Positive Prostate Cancer with Simultaneous Integrated Boost: A Dosimetric Comparison
Source: Adv Radiat Oncol. 2022 Mar 16;7(4):100944. doi: 10.1016/j.adro.2022.100944 (PMC9061255; doi:10.1016/j.adro.2022.100944)
Supplement: Supplementary file 1 [file mmc1.docx]

**Supplemental Table 1: Linear mixed-effects models describing the relationship between nodal displacement and coverage, allowing for per-node and per-patient random effects.**

|  | **Relative coverage loss (%) per 1 mm displacement (95% CI)** | **p-value** |
| --- | --- | --- |
| **Dmean** | -2.1 (-2.5 - -1.6) | < 0.0001 |
| **D95** | -3.2 (-3.7 - -2.7) | < 0.0001 |
| **Dmin** | -3.1 (-3.7 - -2.6) | < 0.0001 |

**Supplemental Table 2: PTV margins according to the van Herk formula predicted to ensure at least 95% coverage for at least 90% of patients.**

| **Direction** | **Systematic error** | **Random error** | **Margin (van Herk) mm** |
| --- | --- | --- | --- |
| **Left** | 1.5 | 1.2 | 4.5 |
| **Right** | 0.9 | 0.6 | 2.6 |
| **Anterior** | 1.4 | 1.2 | 4.3 |
| **Posterior** | 2.5 | 2.3 | 7.9 |
| **Superior** | 3.3 | 1.9 | 9.5 |
| **Inferior** | 0.9 | 0.8 | 2.8 |

**Supplemental Figure 1: Coverage of gross nodal targets for fiducial-based versus pelvic bony-based CBCT registrations. Each data point represents 1 node averaged across the 5 CBCTs for that node.**


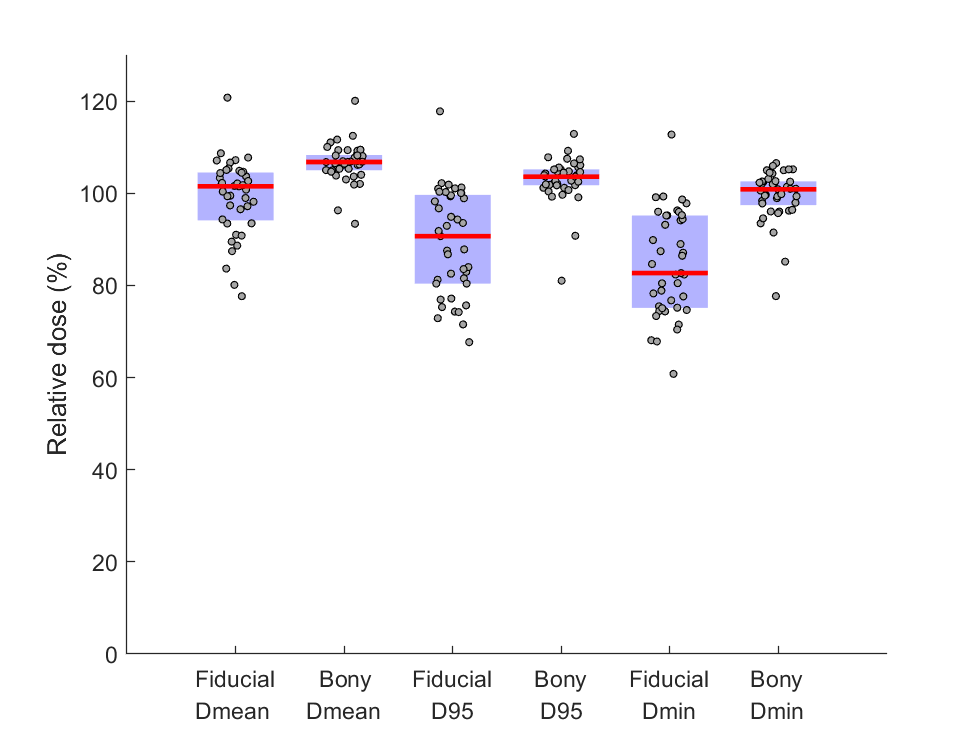


|  | **Fiducial** | **Bony** | **Paired differences**  **(Bony minus Fiducial)** | **p-value** |
| --- | --- | --- | --- | --- |
| **Dmean** | 101.5%  (94.1 - 104.5) | 106.8%  (105.0 - 108.3) | 5.8% (1.5 - 12.2) | < 0.0001 |
| **D95** | 90.7%  (80.4 - 99.7) | 103.6%  (101.7 - 105.2) | 11.2% (4.9 - 22.5) | < 0.0001 |
| **Dmin** | 82.7%  (75.1 - 95.2) | 100.9%  (97.4 - 102.6) | 11.9% (6.3 - 23.7) | < 0.0001 |

* Red bars in the figure and primary values in the table represent medians. Blue patches in the figure and parenthesized values in the table represent interquartile ranges

**Supplemental Figure 2: Coverage of gross nodal targets for fiducial-based versus pelvic bony-based CBCT registrations. Each data point represents 1 patient averaged across all of the nodes within that patient.**


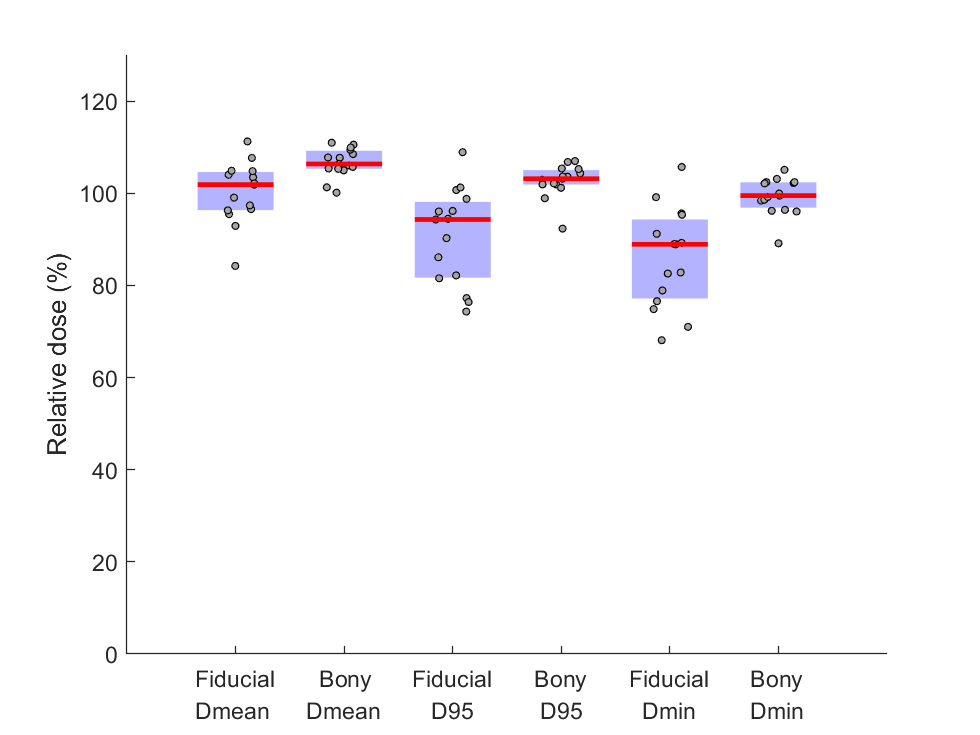


|  | **Fiducial** | **Bony** | **Paired differences**  **(Bony minus Fiducial)** | **p-value** |
| --- | --- | --- | --- | --- |
| **Dmean** | 101.9%  (96.3 - 104.6) | 106.4%  (105.3 - 109.2) | 8.4% (2.3 - 9.8) | 1.22E-04 |
| **D95** | 94.3%  (81.7 - 98.1) | 103.2%  (101.9 - 105.0) | 10.9% (6.5 - 17.9) | 1.22E-04 |
| **Dmin** | 88.9%  (77.1 - 94.3) | 99.5%  (96.9 - 102.4) | 11.2% (9.7 - 18.9) | 1.22E-04 |

* Red bars in the figure and primary values in the table represent medians. Blue patches in the figure and parenthesized values in the table represent interquartile ranges

**Supplemental Figure 3: Amount of Nodal Displacement for Fiducial-Based and Pelvic Bony-Based Registrations Relative to the Planning CT. Each data point represents 1 node averaged across the 5 CBCTs for that node.**


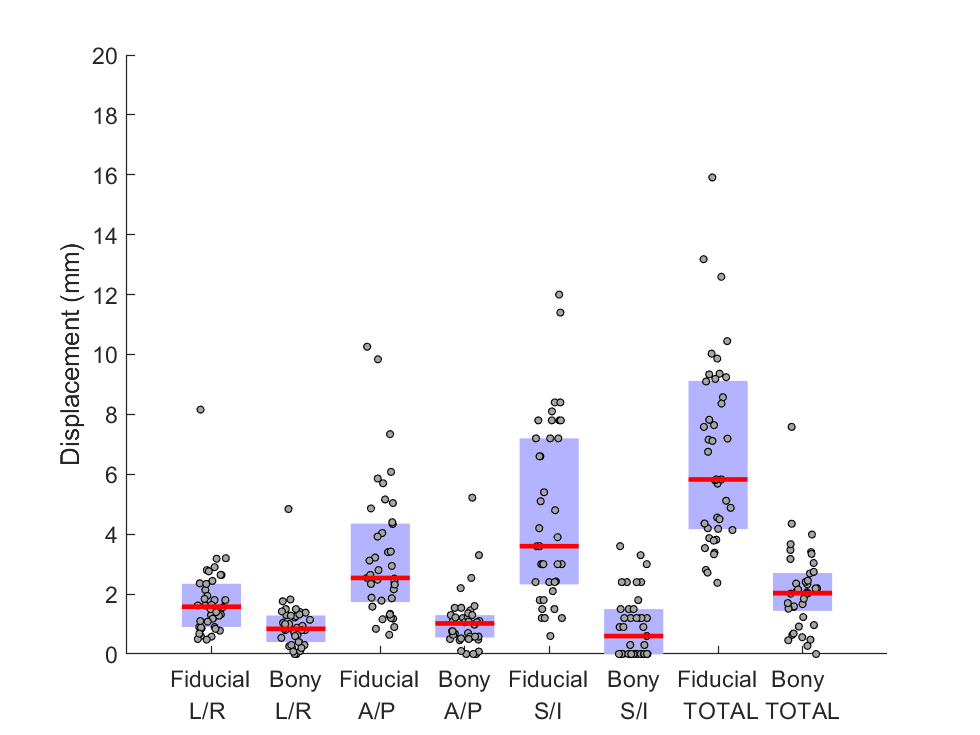


|  | **Fiducial** | **Bony** | **Paired differences**  **(Bony minus Fiducial)** | **p-value** |
| --- | --- | --- | --- | --- |
| **Left/right** | 1.6 (0.9 - 2.3) | 0.8 (0.4 - 1.3) | -0.6 (-1.4 - -0.2) | < 0.0001 |
| **Ant/post** | 2.5 (1.7 - 4.4) | 1.0 (0.5 - 1.3) | -1.8 (-3.4 - -0.6) | < 0.0001 |
| **Sup/inf** | 3.6 (2.3 - 7.2) | 0.6 (0.0 - 1.5) | -3.0 (-6.6 - -1.4) | < 0.0001 |
| **TOTAL** | 5.8 (4.2 - 9.1) | 2.0 (1.4 - 2.7) | -4.3 (-6.7 - -2.6) | < 0.0001 |

* Red bars in the figure and primary values in the table represent medians. Blue patches in the figure and parenthesized values in the table represent interquartile ranges

**Supplemental Figure 4: Amount of Nodal Displacement for Fiducial-Based and Pelvic Bony-Based Registrations Relative to the Planning CT. Each data point represents 1 patient averaged across all of the nodes within that patient.**


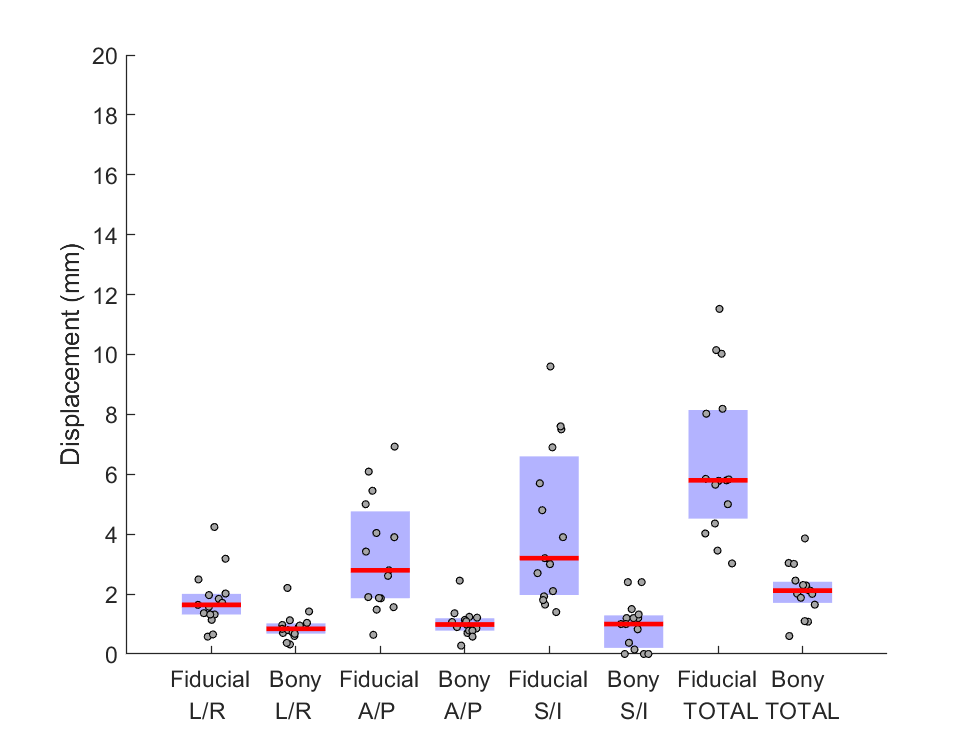


|  | **Fiducial** | **Bony** | **Paired differences**  **(Bony minus Fiducial)** | **p-value** |
| --- | --- | --- | --- | --- |
| **Left/right** | 1.6 (1.3 - 2.0) | 0.8 (0.7 - 1.0) | -0.8 (-1.4 - -0.3) | 1.16E-03 |
| **Ant/post** | 2.8 (1.9 - 4.8) | 1.0 (0.8 - 1.2) | -1.7 (-3.8 - -0.9) | 3.05E-04 |
| **Sup/inf** | 3.2 (2.0 - 6.6) | 1.0 (0.2 - 1.3) | -2.4 (-6.1 - -1.0) | 1.22E-04 |
| **TOTAL** | 5.8 (4.5 - 8.1) | 2.1 (1.7 - 2.4) | -3.5 (-6.2 - -2.8) | < 0.0001 |

* Red bars in the figure and primary values in the table represent medians. Blue patches in the figure and parenthesized values in the table represent interquartile ranges

**Supplemental Figure 5: Relationship between nodal displacement and the magnitude of the bony-to-fiducial vector.**


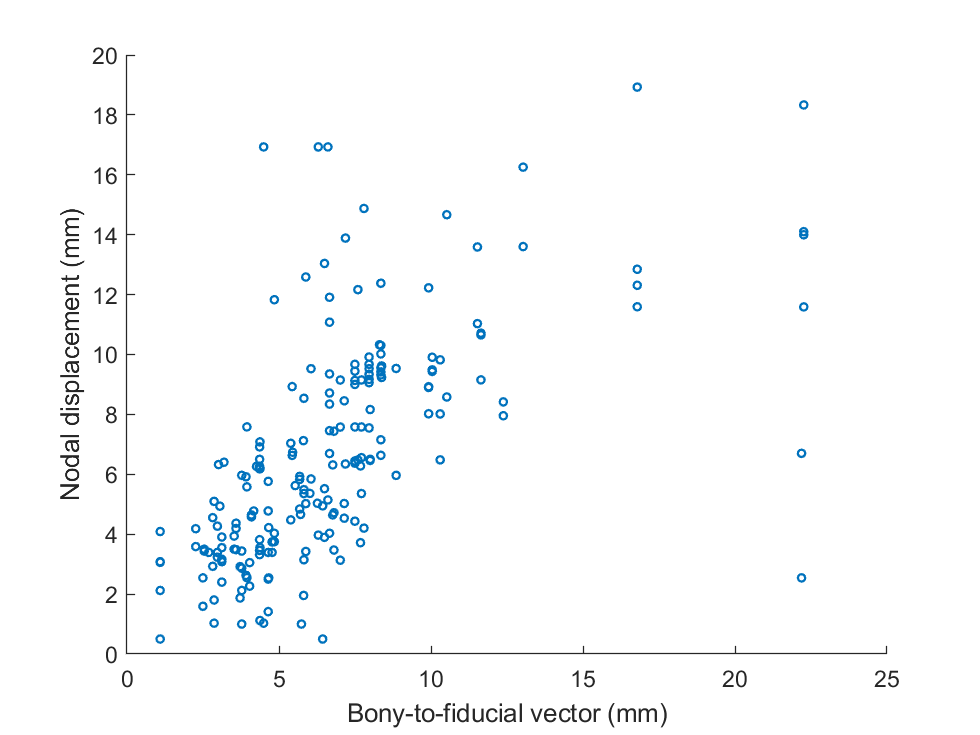


Spearman’s rank correlation: rho = 0.72, p-value = < 0.0001

**Supplemental Figure 6: Cumulative histogram of nodal displacement in each axis on fiducial-based registrations.**

**
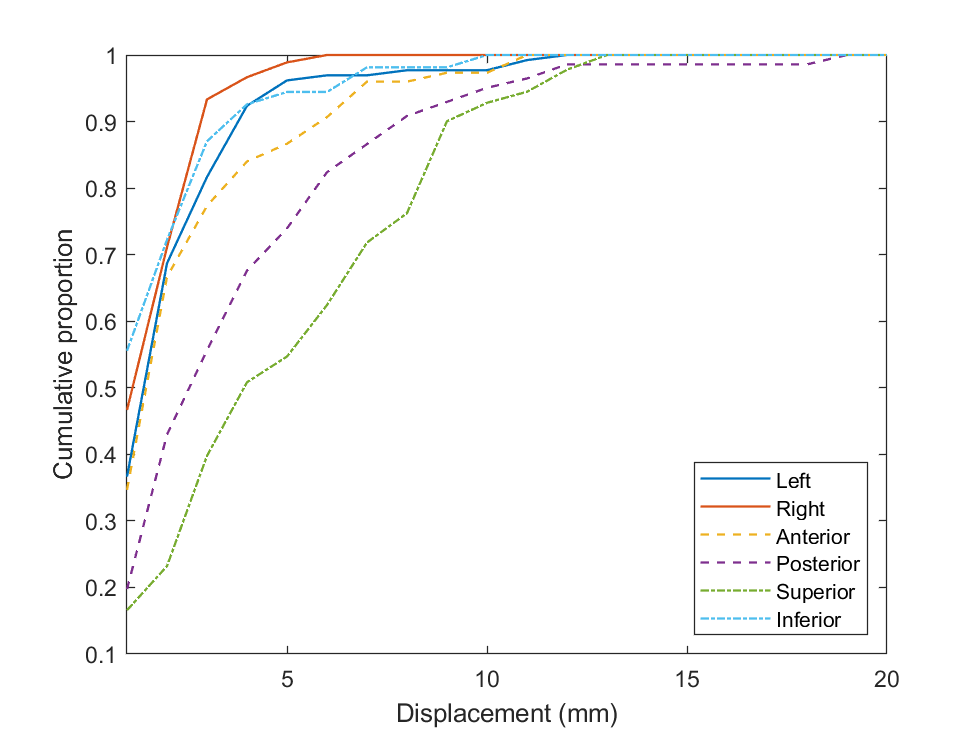
**

* Nodal displacement was significantly different for the anterior vs posterior directions (*P* = .001) and the superior vs inferior directions (P < .0001), but not significantly different for the left vs right directions (*P* = .13)
